# The Braak Stage Specific Gene‐Gene Co‐expression for Alzheimer's Disease in Fusiform Gyrus reveals Important Potential Molecular Biomarkers

**DOI:** 10.1002/alz70855_098313

**Published:** 2026-01-05

**Authors:** Rutvi Dipakbhai Vaja, Darshee Baxi, A.V Ramachandran

**Affiliations:** ^1^ Department of Biomedical Sciences and Life sciences, School of Science, Navrachana University, Vadodara, Gujarat, India; ^2^ Associate Professor & Program Chair, Division of Biomedical and Life Sciences School of Science, Navrachana University, Vadodara, Gujarat, India; ^3^ Navrachana University, Vadodara, Gujarat, India

## Abstract

**Background:**

Alzheimer's Disease (AD) is a progressive neurodegenerative disorder marked by the accumulation of amyloid‐beta (Aβ) peptide, leading to the formation of neuritic plaques and neurofibrillary tangles (NFTs). Braak stages classify the severity of NFTs across different brain regions, helping to track the progression of AD pathology. Recently, the fusiform gyrus has been identified as a critical brain region associated with mild cognitive impairment, which may increase the risk of AD development. However, due to the highly heterogeneous nature of AD, there remains a crucial need to define AD specific gene signatures across various brain regions. This study focuses on identifying alterations in the gene expression profiles of these region‐specific areas, which may provide insight into the selective regional vulnerability that contributes to AD pathogenesis.

**Method:**

In our study, we performed gene co‐expression and differential co‐expression network analyses, as well as gene‐expression‐based prediction, using RNA‐seq transcriptome data from post‐mortem fusiform gyrus tissue samples collected from both cognitively healthy individuals and those with AD.

**Result:**

Our result comprises five gene hubs, namely *USF3, DSE, ID2, BBLN* and *ISF*, which were exclusively present in the AD samples. In fusiform gyrus, five novel candidate genes, namely *NSF, GABRA1, GABRA6, HSPA8* and *IMPDH* were identified co‐expressed across all defined groups, as well as seventy‐four differentially co‐expressed genes. Our approach also identifies braak stage specific hub genes that are specific to each of the AD braak stage. In addition, the plasma membrane transportation and cellular signalling pathways are the most prominent pathway involved in AD processes.

**Conclusion:**

In conclusion, our findings shed new light on the molecular pathophysiology of AD by identifying new genes and biological pathways involved, emphasizing the crucial role of gene regulatory networks in the fusiform gyrus.

